# The promotion of social sustainability by micro-level social inclusion in a multicultural context: literature review and interview evidence

**DOI:** 10.3389/fsoc.2025.1557463

**Published:** 2025-05-21

**Authors:** Qianqian Fan

**Affiliations:** Medill School of Journalism, Northwestern University, Evanston, IL, United States

**Keywords:** multiculturalism, micro-level social inclusion, individual social capital, collective social capital, social sustainability, social capital theory, conceptual model of social sustainability

## Abstract

Drawing on multicultural theory, social capital theory, and sustainability theory, this paper explores social sustainability in a multicultural context. A two-stage research design was employed, consisting of a systematic literature review and interview-based text analysis. In the first stage, 210 papers were systematically reviewed. By extracting conceptual elements, new constructs of micro-level social inclusion, individual social capital, and individual social sustainability were defined. A preliminary social sustainability model—encompassing social inclusion, social capital, and social sustainability—was proposed. Relationship-focused research addressed gaps in existing literature, revealing interactions among these constructs and spurring refinements to the preliminary model. In the second stage, 130 short interview videos (totaling 173 participants) were analyzed. Empirical evidence from these interviews confirms that micro-level social inclusion and individual social capital positively affect social sustainability. The results of this paper are derived from a systematic study of the literature, and the results are verified by the conclusion of interview text analysis. This paper presents an innovative viewpoint by foregrounding micro-level social inclusion and individual social capital in daily life as drivers of social sustainability. Findings demonstrate that micro-level social inclusion fosters individual social capital, which, in turn, is a potent force behind social sustainability. The study thus offers a new research agenda, expands the field of individual social capital research, and provides practical and policy insights for further research and implementation.

## 1 Introduction

In Hofstede’s cultural framework, multiculturalism typically refers to cultural differences and diversity between nations. In practice, geographic factors and immigration often yield diverse local cultures, even within a single country. This diversity can manifest via distinct languages and customs among population groups and becomes more prominent with increased immigration (Evan and Holý, 2023). Hofstede characterizes culture as the “programming of the human mind” that distinguishes one group from another—rarely changing substantially over a person’s lifespan (Hofstede et al., 2014). Consequently, multiculturalism emerges as an influential cultural phenomenon, particularly in immigrant societies.

Although multicultural phenomena have long existed, the formal concept of multiculturalism gained momentum after World War II. From the 1960s to the 1970s, with accelerating globalization and rising immigration, developed countries faced challenges associated with cultural diversity. Multiculturalism subsequently evolved as both a social policy and a philosophical idea, describing a situation in which different groups maintain their unique identities while participating in overarching social institutions (Foulkes, 1995). Multiculturalism is also a conceptual lens used to examine diversity, including factors like ethnicity, religion, and culture, as well as power imbalances (Clayton, 2019).

Although the meaning of multiculturalism can vary across societies and organizations (Verkuyten, 2005), it typically operates at three levels: demographic (cultural diversity and cross-cultural contact), ideological (inclusivity values guiding actions), and psychological (individual attitudes and behaviors toward diversity) (Berry et al., 1977; van de Vijver et al., 2008; Stogianni et al., 2021). Multicultural policies aim to facilitate cross-cultural communication and socio-political integration, uphold diversity, and advance equitable participation across different ethnic and cultural groups (Berry and Colleen, 2016; Ward et al., 2018). Multiculturalism thus includes population, ideology, and policy (Berry, 2013), reflecting notions of inclusion, equality, and empowerment—powerfully shaping societal perspectives in a globalizing world.

Researchers have examined multiculturalism from diverse angles. For instance, increasing immigration has led to more heterogeneous student populations, prompting schools and cultural institutions to adopt strategies that enhance multicultural awareness (Heruti and Yahya, 2024). Meanwhile, multicultural norms and wellbeing intersect in the social domain, where cross-cultural contact, ideology, and policy can generate benefits for immigrants and minorities, ensuring equitable access to education, healthcare, and media coverage (Mateo-Babiano and Fong, 2004). In economics, numerous studies explore relationships between cultural diversity and trade, economic growth, consumer behavior, labor markets, organizational management, and corporate performance (Guiso et al., 2009; Florida, 2002; Luna and Gupta, 2001; Ottaviano and Peri, 2006; Ely and Thomas., 2001; Cox and Blake, 1991; Girdauskiene and Eyvazzade, 2015).

Most multiculturalism research, however, adopts a macro-level perspective. In recent years, researchers have broadened their focus to include more localized issues. Examples include studying the digital divide’s impact on cross-cultural internet consumers (Papadopoulos and Cleveland, 2023), cross-cultural adaptation and culture-sharing on streaming platforms (Li, 2024), and equitable news discourse in a multicultural society (Eranfeno et al., 2024). Policy discussions increasingly highlight cultural adaptation and immigrant wellbeing (Karim and Hue, 2022).

Building on prior work, this paper concentrates on micro-level social inclusion within multicultural settings, addressing how such inclusion promotes social sustainability in everyday contexts (education, healthcare, employment, daily life). Responding to calls for deeper investigation into micro-level social inclusion in existing research, this paper examines how micro-level social inclusion influences social sustainability, particularly via individual social capital.

This research underscores the pivotal role of micro-level social inclusion in individuals’ daily interactions, focusing on how individual social capital (e.g., social networks, norms of reciprocity, and trust) mediates between micro-level social inclusion and social sustainability.

The research in this paper will find the mediating variable between micro-social inclusion and social sustainability, and construct a theoretical model, possibly assuming that the mediating variable is individual social capital. The implicit assumption in this paper is that micro-social inclusion affects individual social capital, and social sustainability is affected through social capital. To this end, this paper designs the working structure of systematic literature research and interview text research.

Conceptual development: this paper proposes a novel perspective on micro-level social inclusion, defining it and examining its relationship with individual social capital.

Practical implications: As globalization and immigration intensify, many countries grapple with cultural diversity and social challenges. Designing inclusion mechanisms at the micro level can be essential for mitigating these challenges.

First, this paper is inspired by research that highlights the importance of multiculturalism. The multicultural context in this paper focuses on everyday multicultural phenomena, investigating how multiculturalism influences individual behaviors, social interactions, identity, and social inclusion in daily life. By analyzing expressions of cultural diversity in day-to-day living, the paper unveils how different cultural elements coexist and accommodate one another in daily life, forming new cultural practices and symbolic meanings (García, 1995).

Second, based on a focus on individuals’ daily lives within a multicultural context—and following extensive reflection after conducting street interviews among the American public—this paper recognizes the close ties between individuals’ daily experiences and multiculturalism. Further, it attempts to explore the links between multiculturalism and social inclusion, and how social inclusion promotes social sustainability.

This research aims to:

Clarify relationships among micro-level social inclusion, individual social capital, and social sustainability through a systematic literature review and an interview-based text analysis.

Identify core elements and indicators of micro-level social inclusion and individual social capital in a multicultural context.

Demonstrate the positive influences of micro-level social inclusion and social capital on social sustainability.

## 2 Conceptual definition and theoretical framework

A review of the existing literature shows that although “social inclusion” is extensively referenced in economics, politics, sociology, and culture, there is no clear consensus on its meaning at the level of daily individual experience. This paper, therefore, introduces the concept of *micro-level social inclusion* as a multidimensional construct. It then develops a theoretical framework that integrates micro-level social inclusion, individual social capital, and social sustainability.

### 2.1 Conceptualizing micro-level social inclusion

#### 2.1.1 Macro and micro levels of social inclusion

Although the literature addresses both macro and micro aspects of social inclusion, the micro dimension is not clearly articulated. Most work focuses on macro-level strategies and policies—such as those designed to increase access to public services for minority or vulnerable groups—while micro-level studies remain relatively sparse. In the research that does address micro-level inclusion, definitions remain broad and rarely delve into individuals’ everyday lives.

Existing work often frames social inclusion in terms of policies and practices that expand access to basic services, thereby equipping marginalized communities with the resources necessary to benefit from social and economic progress (Carneiro et al., 2015). In fact, social inclusion generates a sense of belonging, which scholars recognize as a fundamental human need (Baumeister and Leary, 1995). People routinely engage in day-to-day social interactions to strengthen their self-esteem and sense of belonging, underscoring the fact that social inclusion is not merely about macro-level policies but also hinges on individual choices and behaviors.

In practice, social inclusion is frequently viewed as part of a broader policy agenda, though it also addresses numerous micro-level issues. Originating with the concept of *social exclusion* in 1960s France, social inclusion/exclusion centered on communities marginalized by poverty, unemployment, or social isolation, with the remedy being active efforts to integrate these communities into society (Zeinab Hassan et al., 2022). International organizations such as the United Nations and the European Union subsequently promoted social inclusion in landmark documents (e.g., the *Millennium Development Goals* of 2000 and the *Sustainable Development Goals* of 2015), framing it to eliminate poverty, inequality, and social exclusion (Alaie et al., 2022). As a policy priority in Europe, social inclusion ensures that all individuals participate in social life and enjoy equitable opportunities and rights, thereby shaping a macro-level approach to sustainable development.

Academically, social inclusion is an expansive concept that often overlaps macro- and micro-level domains (Rawal, 2008). In multicultural contexts, social inclusion typically intersects with cultural diversity, emphasizing fair treatment for different ethnic and minority groups in political, economic, and cultural realms (Kymlicka, 1996). This can extend to social justice (Fraser, 2020), economic/financial inclusion (Fonseca and Matray, 2024), and inclusive education (Cooc, 2022). Accelerating globalization, growing immigration, and rapid technological change have all contributed to the complexity of social inclusion, making its definition more diverse in a multicultural setting.

Despite abundant research on social inclusion, key questions persist regarding its micro-level manifestations—especially in multicultural contexts. A more comprehensive understanding of how individuals interact, adapt, and embrace cultural differences in daily multicultural environments is vital for recognizing how micro-level social inclusion influences broader social sustainability. Moreover, empirical studies have yet to provide substantial evidence on how social inclusion manifests in routine behavior within multicultural settings.

#### 2.1.2 Defining micro-level social inclusion

Drawing on the conceptual-attribute extraction approach (Licsandru and Cui, 2018), this paper synthesizes micro-level social inclusion themes from existing definitions by distinguishing between macro and micro emphases. Micro-level social inclusion typically involves promoting individual social participation, implementing welfare policies for fundamental needs (such as nutrition, housing, healthcare, education, and employment), enhancing personal capabilities, providing opportunities, and fostering dignity, acceptance, and support (see Table 1).

**Table 1:** Sample of definitions and levels of social inclusion.

Source	Conceptual description of social inclusion	Level
Collins (2003)	Social inclusion is a theory of how society can be integrated and harmonious. At its simplest, the theory is that if everyone participates fully in society, they are less likely to become alienated from the community and will conform to its social rules and laws.	Micro level
Levitas et al. (2007)	Social inclusion is the opposite of social exclusion and is a complex and multi-dimensional process. Social exclusion relates to the lack of denial of resources, rights, goods, and services, as well as the inability to participate in normal relations and activities available to the majority of people in a society in the economic, social, cultural, or political spheres. It affects both the quality of life of individuals and the equity and cohesion of society as a whole.	Micro level
Sasaki (2010)	Social inclusion should first end the factors that lead to social discrimination and promote the social participation and interaction of individuals.	Micro level
Marino-Francis and Worrall-Davies (2010)	Social inclusion is about each person taking part in society and having control over their own resources. It is also about a community that cares for its members, makes them feel welcome, and is willing to adjust to fit their various needs.	Micro level
Cambir and Vasile (2015)	Social inclusion is understood as the process of dealing with social exclusion and integrating communities into society, and enabling individuals to participate effectively in the economic, social, political and cultural life of mainstream society.	Macro level
Jansen et al. (2014)	We conceptualize inclusion as a two-dimensional concept, which is defined by perceptions of belonging and authenticity	Micro level
Simplican et al. (2015)	Broad conceptions of social inclusion can involve being accepted as an individual beyond disability, significant and reciprocal relationships, appropriate living accommodations, employment, informal and formal supports, and community involvement.	Micro level
Carneiro et al. (2015)	Social inclusion refers to the policies that obtain basic services and build capacity among vulnerable people through opening in order to ensure the benefits of economic growth reach all people.	Non-concrete
Kim et al. (2016)	We regard social inclusion as a process of reaching active participation.	Non-concrete
UN (2016)	Social inclusion is a deliberative process that includes greater participation and embracing equality	Macro level
World Bank Group (2018) and World Bank (2021)	Social inclusion is defined as the process of improving the conditions for individuals and groups to participate in society, as well as the process of enhancing the capacity, opportunities and dignity of vulnerable groups to participate in society in accordance with their identity.	Micro level
Fourat et al. (2020)	Social inclusion can be defined as an ongoing and reflective process of full and participation of all interested social actors, based on respectful interaction between different groups, taking into account simultaneously the community and different classes, ages, genders, etc., regardless of their socio-economic or cultural resources.	Micro level
Lambert (2020)	Social inclusion is commonly used to refer to welfare policies for food, housing, basic health, training, employment and education for unemployed adults who are deprived of mainstream social rights.	Micro level
Xu M. et al. (2024)	Social inclusion is a process aimed at ensuring that individuals and groups are given equal opportunities to participate in the economic, cultural, political and social fabric of society.	Macro level
Bibi et al. (2024)	Social inclusion is defined as the process of ensuring that all members of society have access to the same opportunities and, through policies that act as interventions, ensure the full participation of all members of society to achieve social protection, gender equality and environmental sustainability.	Macro level

From this collective evidence, micro-level social inclusion comprises two key dimensions: (1) *Material conditions* that enable access to social welfare and resources; and (2) *Mental conditions* that provide meaning, belonging, dignity, and identity.

Furthermore, the literature reveals that micro-level social inclusion is intimately connected with individual lived experience, spanning economic, social, cultural, spatial, and policy domains (Bingqin et al., 2024). Economically, it involves ensuring that everyone has equitable access to educational and job opportunities (Amparo and Oyague, 2008). Socially, it nurtures robust interpersonal networks and cultural integration within a diverse society. Spatially, it mandates fair distribution of healthcare and public services. On the policy front, it champions equal voice in decisions affecting individuals’ lives (Scorgie and Forlin, 2019). In fostering social harmony, micro-level social inclusion helps reduce inequality and stabilizes society, thereby enhancing social sustainability (White, 2007).

### 2.2 The conceptual levels of social sustainability

Social sustainability gained traction in the 1970s against the backdrop of heightened concerns about environmental degradation and social equity (Zeinab Hassan et al., 2022). The 1987 Brundtland Report, *Our Common Future*, defined sustainable development as meeting present needs without compromising the future’s capacity to do the same (Alaie et al., 2022). Under the guidance of the UN World Commission on Environment and Development (WCED), policies aimed at social sustainability began to flourish worldwide. Notably, *Agenda 21* from the 1992 Rio Earth Summit positioned social sustainability as core to sustainable development, and the 2015 2030 Agenda for Sustainable Development reinforced this stance by including poverty eradication, quality education, and gender equality within its 17 Sustainable Development Goals.

In the sustainability triad (ecology, economy, and society), the societal component extends beyond natural processes to encompass social processes (Zeinab Hassan et al., 2022). Social sustainability is sometimes dubbed the “cause” of environmental and economic difficulties (Gu and Zhang, 2021), underscoring its pivotal role. However, scholars have yet to reach a unanimous definition of social sustainability (Andrea, 2010). One recurring theme is that social sustainability concerns human interactions and cooperative social processes. For instance, Yiftachel and Hedgcock (1993) describe it as a city’s enduring capacity to support meaningful human interaction, communication, and cultural development, whereas Koning (2001) interprets it as a societal model characterized by justice, equality, and adequate quality of life. Andrea and Tim (2009) consider social sustainability in terms of how individuals attain self-defined development goals.

Social sustainability is thus complex and multifaceted. To clarify its essence, many researchers identify distinct dimensions (Hale et al., 2019). Grounded in the work of Vallance et al. (2011), this paper classifies social sustainability across three levels: individual, relational, and institutional (see Table 2). At the individual level, emphasis is placed on personal wellbeing, core human needs, and quality of life—dimensions crucial to the paper’s central argument. These must be undergirded by relational and institutional supports to flourish.

**Table 2:** Definitions, key terms, and levels of social sustainability.

Source	Conceptual description of social sustainability	Key terms	Level
Yiftachel and Hedgcock (1993)	The sustainability of urban societies as long-term viable environments for human interaction, communication and cultural development.	Culture development; human interaction; communication	Relationship
Sachs et al. (1999)	Social sustainability must be built on fundamental values of fairness and democracy, which means the effective appropriation of political, civic, economic, social, and cultural human rights for all.	Human rights; fairness; democracy	Institution
Polèse and Stren (2000)	Social sustainability is in harmony with the evolution of civil society, creating an environment that fosters the compatible coexistence of cultural and social diversity while promoting social inclusion and improving the quality of life across all population strata.	Quality of life; social diversity	Individual
Harris et al. (2001)	Social sustainability means an equitable distribution of opportunities, full provision of social services including health and education, gender equality, political accountability, and participation.	Gender equality; education; health	Individual
McKenzie (2004)	Social sustainability is a condition for enhancing life within a community and a process by which it can be achieved within a community.	Condition of enhancing life	Individual
Littig and Griessler (2005)	Social sustainability is achieved when social work and related institutional arrangements meet a wide range of human needs, long-term protection of nature and its reproductive capacity, and the norms of social justice, human dignity, and participation are fulfilled.	Protection of nature; social justice; human dignity; human needs	Institution
Andrea and Tim (2009)	Social sustainability concerns how individuals, communities, and societies live together and achieve their chosen developmental models, considering the physical boundaries of their location and the entire planet.	Individuals; communities; life	Individual
Cuthill (2010)	The concept of social sustainability provides a visionary umbrella for exploring ideas of social infrastructure, social capital, social justice, and equity. This relies on participatory governance processes.	Social infrastructure; social capital; social justice; equity; governance	Institution
Dempsey et al. (2011)	Social sustainability must be seen as a dynamic concept that changes over time and place. Changes in local authority service delivery may drive increased social cohesion and interaction.	Dynamics; change; social cohesion; interaction	Relationship
Vallance et al. (2011)	Social sustainability has three components. “Developmental sustainability” involves meeting basic needs, and inter-generational and intra-generational equity. “Bridge sustainability” focuses on behavior change to meet biophysical environmental goals. “Maintenance sustainability” refers to what is socially acceptable or can be sustained socially.	Intergenerational equality; behavior change; basic needs	Individual/Relationship
Murphy (2012)	Many definitions of social sustainability emphasize community participation and economic equity as primary determinants of social sustainability.	Community participation; economic equity	Relationship
Boström (2012)	Social sustainability includes substantive aspects, such as social justice, quality of life, human rights, opportunity, social cohesion, belonging, and health, as well as procedural aspects surrounding participation, communication, governance, and empowerment to achieve developmental goals.	Social justice; quality of life; human rights; opportunity; social cohesion; belonging; health; governance; communication	Institution/Individual/Relationship
Nakanishi and Black (2015)	The term social sustainability can broadly refer to a social state that provides social justice, ensures equality, and affords everyone a decent quality of life.	Social justice; equality; a decent quality of life	Institution/Individual
Opp (2017)	For a city to be labeled socially sustainable, all people, regardless of race, ethnicity, gender, or income level, must have equal access to the benefits of public investment while being able to meet their basic needs.	All people; income; basic needs	Individual
Eizenberg and Jabareen (2017)	Social sustainability includes socially-oriented practices aimed at addressing major social issues to mitigate the risks of climate change and environmental hazards.	Social issues; climate change	Institution
Shirazi and Keivani (2018)	Socially sustainable communities are places where residents highly value and perceive the qualities and interactive practices over a considerable period of time.	Residents; interactive practices	Relationship
Mcguinn et al. (2020)	The core themes of social sustainability involve human wellbeing and equity, access to basic needs, fair income distribution, good working conditions and decent wages, equal rights, inter- and intra-generational justice, access to social and health services, education, social cohesion, inclusion, empowerment, and participation in decision-making.	Human wellbeing; basic needs; fair income; good working conditions; decent wages; equal rights; generational justice	Individual/Relationship
Soltani et al. (2022)	Social sustainability has five indicators: safety, sense of place, and social interaction have been shown to be significantly impacted by spatial configuration, while community stability and community participation show less association with spatial configuration.	Safety; sense of place; participation; community stability; social interaction	Individual/Relationship
Bafarasat (2023)	In summary, social sustainability can be defined as the economic, cultural, and political inclusion of different individuals and groups during development.	Individuals; inclusive; groups	Individual/Relationship
Karakasnaki et al. (2023)	Regarding performance, sustainability means a fundamental shift from efficiency and profit-seeking toward resource preservation and environmental protection. Additionally, employee wellbeing and the quality of working life become part of the social sustainability goals.	Resource preservation; environment protection; employee; quality of working life	Institution/Individual
Sharno and Hiloidhari (2024)	Many enterprises have started to initiate social sustainability frameworks in their business, encompassing social inclusion, resilience, and solidarity.	Social inclusion; resilience; solidarity	Institution/Relationship

From Table 2, it is evident that social sustainability has evolved in definition and scope, forming a conceptual framework with individual, relational, and institutional layers. At the individual level, social sustainability definitions emphasize personal wellbeing, fundamental human needs, and quality of life. This paper views these as the ultimate aims of social sustainability, which is the focal point here—though it requires the support and assurance of social sustainability at the relational and institutional levels.

From these diverse perspectives, one core idea emerges: social sustainability often manifests through daily life and interpersonal engagement. Yet precisely, this *individual* dimension tends to be overlooked, making it the weakest pillar in sustainable development theory and practice (Shirazi and Keivani, 2018). Indeed, some scholars categorize social sustainability in terms of developmental (addressing basic needs and equity), bridge (altering behavior to meet environmental targets), and maintena*nce* (preserving cultural practices and quality of life) (Vallance et al., 2011).

In this context, basic social inclusion is integral to social sustainability. Social sustainability can be succinctly described as “the economic, cultural, and political inclusion of different individuals and groups” within the process of societal progress (Bafarasat, 2023). The pillars of economic, cultural, and political inclusion all substantially influence individual wellbeing and social outcomes, even if they are not always detailed in current research.

### 2.3 Individual social capital vs. collective social capital

Social capital theory clarifies how societal or interpersonal structures support cooperation and mutual benefits (Eizenberg and Jabareen, 2017; Shirazi and Keivani, 2018). Early conceptualizations leaned toward community-wide attributes, though personal dimensions were also implicit. The term “social capital” first appeared in the context of educational literature (Hanifan, 1916), addressing the intangible value derived from people working together to solve daily problems. Bourdieu (1986) and Coleman (2019) later formalized it further: Bourdieu viewed social capital as the sum of resources embedded in durable social networks, while Coleman characterized it as structural elements—e.g., norms, trust, reciprocity—facilitating individual actions within a collective (Coleman, 1988).

Putnam (1992) defined social capital as networks, norms, and trust that promote collaboration and mutual advantage, initially treating it as a collective property. He later shifted toward an individual-level analysis (Putnam, 2001), suggesting that features of social life can motivate cooperation among goal-sharing members of society. Consistent with the theoretical lineage (Portes, 2000; Woolcock and Narayan, 2000; Nan, 2005), this paper views social capital as comprising both individual and collective dimensions (Schwitter, 2020).

According to social capital theory, social networks and relationships are valuable resources for pursuing personal and collective interests (Abane et al., 2024). Following the path of some scholars (Schwitter, 2020; Brunie, 2009; Castiglione et al., 2008), this paper considers social capital to comprise individual and collective dimensions. A systematic literature review can extract the elements of social capital from various definitions, distinguishing whether they belong to personal social capital or collective social capital (Table 3).

**Table 3:** Definitions, key terms, and levels of social capital.

Source	Conceptual description of social capital	Key terms	Level
Hanifan (1916)	Social capital is the connection between individuals in collective action created during problem-solving and daily interactions—an intangible material that applies widely in people’s daily lives through social relationships.	Connection, interactions, people’s daily lives	Collective/Individual
Bourdieu (1986)	Social capital is defined as the total of actual or potential resources related to a lasting network of relationships. It occurs in relational networks and serves as a resource, stimulus, and result of collective action.	Network of relationships, collective action	Collective
Coleman (1988)	Social capital is defined as aspects of social structure that facilitate actions of individuals within the structure and is described as a collective property of the group, referring to community-level social participation, norms of reciprocity, and trust in others.	Collective property, social participation, trust, norms of reciprocity	Individual/Collective
Putnam (1992)	Social capital is defined as networks, norms of reciprocity, and trust that facilitate coordinated and cooperative actions, considered an asset or capacity for collective action at larger social units.	Networks, trust, norms of reciprocity cooperative actions	Collective
Portes (2000)	Social capital can be viewed as a private asset easily used for individual advancement.	Private asset, individual advancement	Individual
Woolcock and Narayan (2000)	Social capital involves trust, care, empathy, or willingness to go beyond self-interest for mutual benefit.	Trust, care, empathy, willingness	Individual
Putnam (2001)	Social capital usually refers to features of social life that promote cooperation and coordination between individuals with common goals.	Social life, cooperation, and coordination between individuals	Individual
Woolcock (2001)	Social capital can be interpreted as “bonding,” “bridging,” and “linking” social capital.	Bonding, bridging, linking	Collective
Lochner et al. (2003)	Social capital is often viewed as a collective property where social structures (interpersonal trust, norms of reciprocity, and mutual assistance) serve as resources for collective action.	Collective property, social structures, collective action	Collective
Nan (2005)	Social capital is valuable resources acquired by individuals or organizations through the establishment and accumulation of social networks, including trust, cooperation, reciprocal support relationships, and information flow.	Individuals, organization, social networks, trust, cooperation, reciprocal support	Individual/Collective
Tirmizi (2005)	Social capital refers to aspects of social structure that provide members with resources that can be used to pursue their interests.	Social structure, members, Interests	Individual
Nan and Erickson (2008)	Social capital is defined as “resources embedded in social networks.”	social networks	Individual/Collective
Aldrich and Meyer (2015) and Chen and Volker (2016)	Social capital is widely used to explain both individual and community development.	Individual, community	Individual/ Collective
Shiell et al. (2020)	Social capital is defined as characteristics of social organizations, such as civic participation, norms of reciprocity, and trust in others, which promote mutually beneficial cooperation.	Social organization, trust, Participation, norms, Cooperation	Individual/ Collective
Tahlyan et al. (2022), MacGillivray (2018), Kant and Vertinsky (2022)	Social capital has a multidimensional nature. The intensity of social relations among social citizens, the frequency of social resource exchanges, perceived social equity and justice, and the degree of solidarity and mutual trust are core elements of the concept of social capital.	Social relations, social citizens, social resource, social equity, justice, trust, solidarity	Collective/ Individual
Utsumi and Muradi (2024)	Social capital is conceptualized as inherent characteristics of social networks that connect individuals or groups and improve social efficiency through cooperative behavior.	Social networks, social efficiency, cooperative, individual, groups	Collective/Individual
Xu Z. et al. (2024)	Social capital refers to the state and characteristics of close connections between individuals, groups, societies, and even nations and other social entities.	Connections between individual and groups, societies	Collective/Individual

Table 3 illustrates the distinction between personal and collective aspects of social capital. Individual social capital involves personal relationships, trust, empathy, and reciprocal support, while collective social capital resides at the societal or group level (Castiglione et al., 2008). From this perspective, individual social capital—encompassing family ties, friendships, reciprocal expectations, and interpersonal trust—closely aligns with micro-level social inclusion: both concepts focus on the personal experience of social interaction and belonging. Preliminary Conceptual Model of Social Sustainability.

Only a limited number of studies employ a conceptual model to explore social sustainability directly. In an early example, Assefa and Frostell (2007) proposed a model linking ecological sustainability to social and economic sustainability through technology. Some researcher later introduced a framework emphasizing community decision-making processes, highlighting how community transformation needs social capital as a pathway to social sustainability.

Recent research has continued this modeling trend, often with a technological emphasis. Tahlyan et al. (2022), for instance, presented a five-subsystem framework (socio-political, socio-cultural, socio-institutional, socio-economic, and socio-environmental) to assess building systems. In another example, Arpaci et al. (2022) incorporated personality traits into the UTAUT2 model to analyze social sustainability in the metaverse, showing how constructs such as performance expectancy, habit, and social influence shape social sustainability at the micro level.

**Figure 1:**
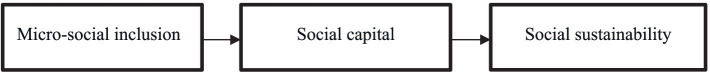
Preliminary conceptual model of social sustainability.

Against this backdrop, the present paper proposes a *preliminary conceptual model* highlighting three main variables (see Figure 1): micro-level social inclusion, social capital, and social sustainability. Prior scholarship suggests that social capital is a conceptual foundation for social sustainability (Cuthill, 2010). As a proxy for societal relationships (Kamaludin et al., 2021; Kamaludin, 2023), social capital influences social sustainability, which ultimately depends on human behavior and interaction. Micro-level social inclusion—encompassing equal opportunities in employment, security, healthcare, and overall quality of life (Pazhuhan et al., 2023)—is hypothesized to exert a positive effect on social capital. Additionally, empirical findings show that social capital’s structural dimension (social networks, interpersonal trust, reciprocity) can fully mediate this relationship (Su et al., 2024).

In summary, on the basis of the definition of relevant concepts, this paper has obtained some verification from the study of street interview texts on the basis of the systematic study of the literature on how social inclusion at the micro level affects social sustainability in the everyday environment (education, health care, employment, and daily life). Social inclusion at the micro level lays the foundation for long-term social stability and development through equitable resource distribution and increased individual wellbeing.

## 3 Methodology and research design

The research paradigm here combines interpretive empiricism and theory, emphasizing understanding and interpreting phenomena in the social domain. Under this paradigm, the core assumption is that a multicultural context and micro-level social inclusion have a positive impact on individual social capital, which in turn has a positive impact on social sustainability, and that multicultural context and micro-level social inclusion indirectly impact social sustainability through individual social capital. This study focuses on interpreting definitions of related concepts and constructing a conceptual model, thereby demonstrating the relationships among a multicultural background, micro-level social inclusion, individual social capital, and social sustainability.

Qualitative methods are employed, including literature analysis, text analysis, and discourse analysis. As an exploratory study, it does not rely on a specific case but on a systematic literature review combined with short street-interview videos collected by the author to elucidate, from a broader perspective, the influential variables of social sustainability. Therefore, this paper develops a two-phase research design (Table 4).

**Table 4:** Two-phase research design.

Procedure	Phase 1 Systematic analysis of the literature	Phase 2 Text analysis from short video of random street interviews
Approach	210 papers via Elsevier and Google Scholar Systematic and comprehensive literature review	173 respondents from all walks of life in street of New York City and Jersey City in U.S.
	Extract subject words from papers	Original interview text from 130 Short videos on YouTube
Purpose	Identify indicators of related concepts within the literature	Investigate evidence related to conceptual dimensions
	Test the proposed preliminary conceptual model	Test the proposed modified conceptual model
Method	Manual content analysis, Voyant Tools	Manual content analysis, Voyant Tools
Outcomes	Extracted factors of SI, SC, SS and their related context	Evidence from interview texts
	Confirmed the proposed preliminary conceptual model as logical	Understand complex practical phenomena
	Form a modified conceptual model	Development of full conceptual model of social sustainability

SI, social inclusion; SC, social capital; SS, social sustainability.

In Table 4, Phase 1 is a systematic analysis of existing literature aimed at identifying the themes, dimensions, or elements of multicultural context, social inclusion, social capital, and social sustainability, as well as discovering examples of relationships among these variables. This leads to the initial and revised conceptual frameworks of social sustainability. Phase 2 analyzes random street-interview short videos, collecting and screening interview texts. Through text analysis and discourse analysis, relevant keywords are extracted, providing empirical evidence for Phase 1’s findings.

### 3.1 Phase 1

The main purpose of Phase 1 is to refine and revise the detailed elements and interrelationships in the initial social sustainability conceptual model presented in Figure 1. During the literature review process, papers were retrieved from the Elsevier database using the keywords “social inclusion,” “social capital,” and “social sustainability,” restricted to review articles and research articles with no limits on Subject areas or publication date, supplemented by Google Scholar searches. Following a principle of data saturation, searches stopped when no further exact keyword matches were identified. From 387 papers, 210 were carefully selected after excluding single quantitative studies that did not meet the research requirements.

During the study, a manual coding approach was used to develop a set of terms for each conceptual theme (Curcija et al., 2019), followed by thematic keyword extraction. Based on a limited number of references, an exploratory analysis of the relationships among the key variables (micro-level social inclusion, social capital, social sustainability) in the preliminary social sustainability model was then conducted. Finally, a more comprehensive conceptual framework was developed, and the social sustainability model was revised.

### 3.2 Phase 2

**Figure 2:**
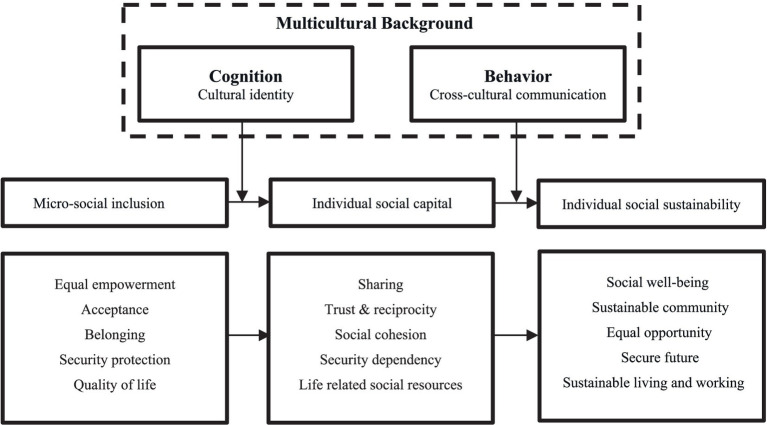
A revised conceptual model of social sustainability.

The main goal of Phase 2 is to validate the elements and relationships of the revised social sustainability conceptual model (Figure 2 in the original paper). Data for text analysis come from short random street-interview videos. Random interviews were used to explore individuals’ subjective experiences and interpretations of specific phenomena, seeking to understand people’s genuine thoughts and feelings under natural daily-life conditions—aligning with the study’s focus on micro-level social inclusion.

The author conducted the interviews from February to October 2023 in New York City and Jersey City, using theme-word prompts and semi-structured open-ended questions. About 130 two-minute videos were produced and uploaded to YouTube (see Appendix A), featuring 219 participants. After filtering out incomplete data and selecting respondents whose answers were relevant to the research theme, 173 participants were retained. Demographically, the oldest participant was 82, the youngest 15, with 83 males and 90 females from a wide range of professions. Their annual incomes spanned about $100,000–$300,000, $60,000–$90,000, and below $60,000, and their educational backgrounds ranged mostly from university/college to a small portion with only high school education. Their ethnic backgrounds reflected cultural diversity, including African American (32), White (86), Asian (15), Hispanic (15), and Indian (25). This study focuses on the breadth of respondents and content analysis of interviews, so the demographic characteristics are not analyzed further.

These themed prompts and open-ended questions yielded rich text data, achieving data saturation in collecting and collating interview transcripts. The transcripts were manually recorded for management and subjected to manual content analysis to refine conceptual definitions. Coding identified primary and subcategories. Guided by an interpretive empiricist paradigm, reliability and validity in qualitative research were approached through credibility, transferability, dependability, and confirmability (Lincoln and Guba, 1985). During interviews, the “credibility” principle was observed by achieving data saturation (Eisner, 2017) and frequently paraphrasing participants’ descriptions to ensure no difference in meaning between the interviewer and interviewees (Creswell, 2007). Consistency in the same measurement tools over different times and places was also maintained. Moreover, the interview’s theme words matched the conceptual definitions being studied, fulfilling the “confirmability” criterion.

## 4 Results

### 4.1 Phase 1 results

Phase 1 results come from a systematic literature review of 210 selected papers related to social inclusion, social capital, and social sustainability. Manual methods were supplemented by Voyant Tools, yielding a systematic analysis to identify research themes in these works. Based on thematic analysis, conceptual elements and levels in the literature were identified, refining, and revising the preliminary social sustainability model. With further reference to literature clues, the conceptual framework was enriched and logical validation and revision of the preliminary social sustainability model were completed.

#### 4.1.1 Analysis of conceptual dimensions and elements

##### 4.1.1.1 Micro-level social inclusion

Literature on social inclusion spans diverse fields, including social sciences, public policy, education, urban planning, and health. Generally, social inclusion includes four dimensions: economic, social, cultural, and political (Atkinson and Marlier, 2010). These dimensions appear in policy measures related to the economy, education, poverty, health, development, equity, and immigration. While these four dimensions of social inclusion typically refer to macro-level perspectives, some literature also confirms micro-level social inclusion, such as gender equality (Kabeer, 2016), housing exclusion (Baptista and Marlier, 2019), subjective feelings of inclusion (Licsandru and Cui, 2018), enjoyment of video games (Bowmanm et al., 2015), lack of income or household goods (Cambir and Vasile, 2015), and unequal food distribution (Fourat et al., 2020).

Building on existing studies, this paper interprets micro-level social inclusion as an emphasis on material or spiritual conditions that can enhance personal wellbeing. From a literature-based perspective, nine elements of micro-level social inclusion were identified: Acceptance, Belongingness, Empowerment, Equality, Respect, Ethnic self-referencing, Mobility, Community engagement, and Gender empowerment. These nine elements fall under the scope of micro-level social inclusion because, in daily life and work, they often represent individuals’ fundamental social needs.

##### 4.1.1.2 Individual social capital

Research on social capital suggests it is a concept describing social relational forces at personal or collective levels (Peluso et al., 2024). Despite varied definitions, social capital is typically regarded as a collective property where social structures—like interpersonal trust, reciprocity norms, and mutual assistance—serve as resources for collective action (Kawachi et al., 1997; Lochner et al., 2003). Meanwhile, social capital also denotes resources or benefits individuals gain from connections with others, linking individual behaviors to organizational functions and then to society overall (Coleman, 1988, 1994; Kawachi et al., 2008; Putnam et al., 1992). In other words, social capital comprises both individual- and collective-level attributes, acting as an intermediary driving social sustainability. Social capital’s three fundamental dimensions—social networks, social trust, and social norms—promote cooperation and mutual benefit among individuals, with high social capital levels leading to more prosperous, resilient communities (Putnam et al., 1993).

Individual social capital is one form of social capital, manifested more concretely under the three fundamental dimensions of social capital. The structural dimension of social networks—both strong ties (family, relatives, close friends) and weak ties (neighbors, acquaintances)—constitutes individual social capital (Yanjie et al., 2012; Granovetter, 1973). The trust dimension is the core of social capital. Unlike the generalized trust that reflects an entire society, particularized trust, limited to family relationships, friendships, and contractual relationships, is part of individual social capital (Weimin and Yucheng, 2002). The reciprocity-norm dimension likewise appears as individual social capital. Whether as a support system or a constraint mechanism, reciprocity manifests in individual social capital. Instrumental support brings an abundance of available resources that help improve one’s life (Lin et al., 1999). Emotional support meets basic psychological needs like care, companionship, and sharing, enhancing self-identity (Xiumei Zhu et al., 2013). As a constraint mechanism, shared values and behavioral norms enable individuals to better conform to collective standards and enjoy a collective identity and sense of belonging (Yongqian, 2016).

From the literature, eight elements of individual social capital emerge: Community Governance, Neighborhood and Schools, Trust and Networks, Family Social Networks, Health and Wellbeing, Elderly and Loneliness, Social Cohesion, and Community Development (Table 5). These elements are especially salient in personal development and reflect subjective social capital.

**Table 5:** Elements of micro-social inclusion and individual social capital.

Concept	Elements	Concept	Elements
Micro-social inclusion	1. Acceptance	Individual Social capital	1. Community governance
	2. Belongingness		2. Neighborhood and schools
	3. Empowerment		3. Trust and networks
	4. Equality		4. Family social networks
	5. Respect		5. Health and wellbeing
	6. Ethnic self-referencing		6. Elderly and loneliness
	7. Mobility		7. Social cohesion
	8. Community engagement		8. Community development
	9. Gender empowerment		

##### 4.1.1.3 Individual-level social sustainability

From the literature review, social sustainability integrates various conceptual elements such as quality of life, social cohesion, sustainable communities, livability, and social wellbeing (Talan et al., 2020). Key themes of social sustainability include human wellbeing, access to basic needs, fair income distribution, decent working conditions, equal rights, intergenerational justice, availability of health and education services, social cohesion, inclusion, empowerment, and participation in decision-making (Mcguinn et al., 2020). In essence, social sustainability often revolves around interconnections between unique life opportunities and institutional structures, or between personal behavior and the environment (Jarvis et al., 2001).

Specifically, social sustainability indicators can be categorized into individual, relational, and institutional levels (Hale et al., 2019). Various forms and keywords describing social sustainability in the literature can be attributed to these three layers. Social sustainability is undoubtedly broad; repeated attempts have been made to summarize its indicators differently. One approach highlights three components: “developmental sustainability” (meeting basic needs, ensuring intra- and inter-generational equity), “bridge sustainability” (changing behavior to meet environmental goals), and “maintenance sustainability” (maintaining existing cultural habits and quality of life) (Vallance et al., 2011). Developmental sustainability includes fair incomes and access to housing, goods, services, and employment (Sachs, 1999). Hence, social sustainability can be used both broadly to refer to systems providing social justice and equality and more narrowly to a social state ensuring individuals a dignified quality of life.

Unquestionably, social sustainability is an expansive concept. Its fundamental dimensions—social equity, social cohesion, quality of life, and participation—make up the social pillar of sustainable development, also serving as an analytical framework for relevant policies (Murphy, 2012). This paper aims for an in-depth analysis of a particular aspect. Social sustainability includes substantive concerns—such as social justice, quality of life, human rights, opportunities, social cohesion, belonging, and health—as well as procedural concerns like participation, communication, governance, and empowerment (Boström, 2012).

In the view of this paper, individual-level social sustainability more strongly reflects substantive aspects of social sustainability. Achieving social sustainability overall begins with satisfying the personal basic needs on which human wellbeing is founded. Therefore, this paper extracts a set of individual-level social sustainability elements from the literature, confirming that this layer is real and concrete (Table 6).

**Table 6:** Sample of individual social sustainability factors.

Source	Factors	Literature theme
Sachs (1999)	Equitable income distribution, employment, equitable access to resources and social services	Dimensions of social sustainability
Spangenberg (2004)	Income, communication and participation, education, social contacts	Sustainability indicators
Choguill (2008)	Feeling of belonging, mutual support, safety, interpersonal relations among the neighborhood	Sustainable neighborhoods
Bramley et al. (2009) and Weingaertner and Moberg (2011)	Affordable housing, satisfaction with home, access to facilities and amenities, sense of place	Urban social sustainability
Mani et al. (2016) and Ahmadi et al. (2017)	Health and wellbeing, education and training, fair distribution (income, employment), housing, participation, human dignity	Smart cities
Hale et al. (2019) and Montalbán-Domingo et al. (2021)	Health, safety, equality, education, training, gender diversity, work conditions, safety and health	Social sustainability of supply chain
Nejada et al. (2021)	Increased income for all groups (e.g., age, sex, ethnicity, religion, class), improved welfare (e.g., wages, benefits, work hours)	Social sustainability indicators
Arpaci et al. (2022)	Job stability, work and occupational health and safety performance, social benefits and security, professional ethics nondiscrimination and equal opportunities, technical training, fair	Social sustainability in public-works procurement
Taherkhani (2023)	Employment, services/facilities, health, equality, safety, social acceptance	Social sustainability of technology management
Sugandha et al. (2022)	Performance expectancy, price values, habits, effort expectancy, enjoyment motivations	Social sustainability of the Metaverse
Soltani et al. (2022)	Sense and perception, quality of life, education and knowledge, job and employment, future, wages and income distributions	Social sustainability assessment framework
Hofstad (2023)	Equality, quality of life, diversity, citizen occupational health, education	Social sustainability of density
Mouratidis et al. (2024)	Sense of place, social interaction, community participation, community stability, condition of being protected from harm	Housing, schools, public transport, health, quality of life, trust, norms

#### 4.1.2 Relationship analysis among social inclusion, social capital, and social sustainability

Analyzing the relationships among social inclusion, social capital, and social sustainability reveals few direct references. Some literature examining best practices in social sustainability for European cities specifically addresses social inclusion, social capital, and community participation in urban regeneration (Andrea and Tim, 2009), indicating that social inclusion, social capital, and community engagement are crucial for sustainable urban development.

##### 4.1.2.1 Social inclusion affects social sustainability

Existing studies tend to discuss their positive interaction when investigating the assessment dimensions and approaches for achieving social sustainability. Some works rank social inclusion among core dimensions of social sustainability—alongside social equity, social cohesion, quality of life, and social participation (Davidson, 2010; Dempsey et al., 2011; Boström, 2012; Eizenberg and Jabareen, 2017)—and verify these indicators (Boström, 2012). Social inclusion thus emerges as a foundational metric in evaluating social sustainability. Other research views social inclusion as a critical tool for realizing social sustainability, especially efforts addressing poverty, educational equity, and gender equality (Sachs et al., 2019). Some studies, using a new framework like “doughnut economics,” explore economic inclusion as vital to social sustainability (Raworth, 2017).

Social inclusion fundamentally aims to remedy social exclusion. One major threat to social sustainability is long-term poverty driven by unfair distribution, arising from social and political exclusion (Friedmann, 1996). Achieving social sustainability must tackle exclusion, as exclusion obstructs access to resources and opportunities needed for improving life (M-Keivani et al., 2020). Clearly, social inclusion, including maintaining cultural diversity, is vital to social sustainability (Andrea, 2010).

Practical examples can be found in numerous domains—fair energy distribution, community governance of protected areas, inclusive governance via social contracts—demonstrating social inclusion’s key role in social sustainability (Sovacool and Dworkin, 2015; Bennett and Dearden, 2014; Hickey et al., 2016). Without question, implementing social justice and equality via social inclusion significantly enhances social sustainability (Missimer et al., 2017). As a result, some scholars emphasize that social inclusion must be prioritized in urban sustainability planning to promote fairness, inclusion, and democratic participation (Fainstein, 2013).

##### 4.1.2.2 Social inclusion promotes social sustainability through social capital

On one hand, achieving social inclusion in a multicultural context faces cultural differences and cross-cultural barriers. The trend of “super diversity” in social development can undermine mutual trust, decreasing social inclusion’s impact on social sustainability (Putnam, 2007; Vertovec, 2007). On the other hand, social inclusion can leverage social capital—expanding social networks, building interpersonal trust, and eliminating relational barriers to social sustainability.

According to social capital theory, social capital is an asset or capacity for action, enabling coordination and cooperation for mutual benefit among those with a common goal (Putnam, 1992, 2001). As a resource that can be deployed, social networks, trust, and reciprocity can either foster or hamper social inclusion (Cheong et al., 2016). Empirical research has shown that the structural dimension of social capital (social ties, social participation, social cohesion) and its cognitive dimension (trust, reciprocity) play regulatory roles in individual psychology and behavior (Pérez-Sastré et al., 2024). Both macro and micro forms of social capital—spanning social networks, trust, reciprocity, government governance, and local management—serve as mediating bridges in addressing poverty, individual apathy, and fostering social relations and cooperation.

Social inclusion and social sustainability share a common end goal. Bridge sustainability aims at behavioral change to fulfill environmental goals, and maintenance sustainability seeks to preserve social and cultural patterns under socioeconomic changes (Vallance et al., 2011). Both require concrete action and practice. Social capital is a robust asset for such action. Thus, social inclusion can advance social sustainability by influencing, building, and amplifying social capital. Through social interaction, expanded communication, and information sharing, social capital can affect knowledge, awareness, attitudes, and ultimately human behavior (Xu Z. et al., 2024).

#### 4.1.3 Revised conceptual model of social sustainability

The outcome of the literature review is a fully developed social sustainability conceptual model (Figure 2). Compared with the preliminary model (Figure 1), the revised version introduces four variables—multicultural background, micro-level social inclusion, individual social capital, and individual social sustainability, to show:

a. Social sustainability development is fundamentally about meeting people’s basic needs. Individual-level social sustainability is the foundation of social sustainability as a whole. b. Micro-level social inclusion, individual social capital, and individual social sustainability correlate closely with personal needs. c. Under a multicultural background, cognition and behavior affects individual-level social sustainability.

Under the three-goal paradigm of social sustainability, developmental sustainability correlates with cognition and needs, aiming to address basic needs, foster social capital, and uphold social justice (Vallance et al., 2011), whereas bridge sustainability and maintenance sustainability relate to behavior. By linking social sustainability to human needs, cognition, and behavior, this paper provides a new perspective in the literature. Among factors affecting social sustainability, personal traits have garnered scholarly attention: in the Big Five personality framework, agreeableness, neuroticism, and openness significantly influence social sustainability in virtual social interactions, with habit being the strongest predictor (Arpaci et al., 2022). Additionally, social inclusion that generates a sense of belonging meets a fundamental human need (Baumeister and Leary, 1995) and fosters social networks, trust, and reciprocity norms.

In Figure 2, the revised conceptual model of social sustainability considers the multicultural background and incorporates the cognitive and behavioral aspects from the S-O-R (stimulus-organism-response) theory (Mehrabian and Russell, 1974) as moderators. Based on Maslow’s hierarchy of needs (Abraham, 1943), five key elements each are identified for micro-level social inclusion, individual social capital, and individual social sustainability: physiological needs, security needs, social needs, esteem needs, and self-actualization needs. This revised model forms a new conceptual framework at the micro level for social sustainability, contributing a key result of the literature review (Figure 2).

### 4.2 Phase 2 results

Phase 2 results derive from analyzing short video transcripts. After filtering, interview transcripts from 173 participants were selected. Semi-structured questioning was conducted randomly on the streets of New York City and Jersey City, covering a wide array of responses. Transcripts were subjected to qualitative text analysis methods—manual coding, thematic analysis, and sentiment analysis—to extract key themes relevant to this study. Directed content analysis was employed (Hsieh and Shannon, 2005), classifying the textual content according to the research objectives.

#### 4.2.1 Revised conceptual model of social sustainability

The first step was to organize the original transcript data and conduct comparisons and abstractions to form basic categories. Using manual open coding, 12 preliminary categories were identified, and participants’ attitudes were analyzed. The initial categorization provides insight into the day-to-day attitudes and core demands of the general public regarding issues related to social sustainability (Table 7).

**Table 7:** Preliminary coding analysis of original text.

Sample of original text data	Category	Attitude
Family, and personal time, and mental health is important.	Family and marriage	Positive
I would not risk that for any money, everyone needs family.		Positive
You get to make a family. I want get children.		Positive
Yes, actually I married. We can have a good life together.		Positive
I, myself, would love to be married. But I do not think it is necessary.		Negative
Marriage is actually a very very essential.		Positive
…		
For my job in general? I feel like a good balance.	Work-life balance	Positive
My hours are unusual. So, it does not line up with a lot of other people. I spent time with my children very once in a while. I get to see friends but not terribly often.		Negative
We do not actually get paid time off. You know the service industry. Especially need work-life balance. It is hard to find work-life balance.		Negative
…		
I enjoy work very much.	Job and retirement	Positive
Most expensive place here, NYC. I do not plan to retire here NY. I want to retire where not hot, not cold, the quality is good, living very cheap.		Negative
To save to retire, I save 25% my salary. I try to save 30%.		Positive
Do not overwork.		Negative
Maybe I have contributed more to like my 401 k.		Positive
I like the four-day work.		Positive
A four-day work would be a blessing.		Positive
…		
For couple, they both have their salaries and one account. If they have a house together or anything like that, all their things they share together.	Sharing	Positive
Financial advisors are helpful.		Positive
If they think the level is safe then you would hope they are doing the right thing (nuclear wastewater). I do not have enough information.		Negative
You have someone you can share things with.		Positive
Everybody should be allowed to do their own thing, should be shared.		Positive
…		
Beautiful ring, in our discussion it matters most what it meant, the commitment was the important thing.	Trust and Charity	Positive
Do not trust online product picture.		Negative
I give a percentage to charity.		Positive
Running charity feed homeless.		Positive
…		
Friendship is rarer than money. Betraying friends for money? No.	Friendship and relationship	Positive
Friendship before money, life is about so much more.		Positive
For money, yes. I can always find new friends.		Neutral
I love my best friend. I could not do that to her.		Positive
Leave friend for money? Yes. I give money to him some of like 5 million.		Negative
I think I value her friendship.		Positive
I give him money I feel like a few million makes up for our friendship.		Neutral
I might even be skeptical about that money is important.		Positive
Friends are just more important to me.		Positive
I have a great mentorship.		Positive
You want to be resourceful with relationship, it is something that needs to be spoken about more when somebody else is involved in your life.		Positive
…		
I feel like in North America, it is better workplace, gender equality is better than some of the countries. And specifically for my firm, we have pretty nice policy for women, for pregnancy leave and for unlimited paid time off.	Equality	Positive
I feel like there still is a structural unfairness or inequality.		Negative
I feel like it is hard to make a conclusion on the whole structure of all of women in the world.		Neutral
You start seeing woman as a leader beyond just chief marketing officer. You start seeing that equality is really at play.		Positive
And a lot of times even though we say like diversity or equality across women.		Positive
Definitely, equal, I work with animal.		Positive
…		
I am blessed with my job because we have good benefits as teachers. I’ve worked in the past with like food service and different service industry type things.	Wellbeing and health	Positive
I think that is I mean same thing compare to like a horrible job with no pay off, this is good but it is hard to say.		Neutral
I have 401 k, pension plan that includes health, dental, med, everything in between I get great benefits.		Positive
Health insurance, retirement pension, we do not have much, do not make that much.		Negative
401 k is your saft net, a 100% recommend it.		Positive
401 k is good idea to save for the future, seniors have their own money.		Positive
Medical, it is not great.		Negative
I do not have 401 k, so I save more, not spend most money.		Negative
401 k is not necessary. You just to restrict what you do, saving a sort of.		Negative
…		
The rent is 1,350$ per month, that is high. But I am price pretty well.	Rent, price, and housing	Positive
The 1,200$ per month, a little bit high.		Negative
The 3,000–5,000$ per month, very expensive.		Negative
Yes. Rent very expensive to live in. The food is expensive, car is payments, everything is a little bit more expensive here.		Negative
Living expense are high.		Negative
Rent always keeps going up, sucks.		Negative
Rent definitely high, a lot of inflation minimum wages not being up to with the living costs.		Negative
My rent is ok, 2,500$ per month. I am getting my money’s worth. I live in nice building.		Positive
It is more than 30% increases within a year.		Neutral
Everything here is horribly expensive when I come to pet.		Negative
Yes, I want to buy a housing. I have unrealistic expectation.		Negative
Gas price is too much.		Negative
College, my family support me and finance aid 3,000$, it is not so much, but it is something too expensive, compare other countries where it is free, here it is too expensive.		Positive
My dream house would be in the woods, probably upper side NY with the woods, the mountain, lake.		Positive
1,200$ per month, a little high, that is why we decided to go for a home instead of rent.		Negative
I live in a nice building. It is got a concierge, comfortable. The problem is with gentrification.		Negative
Dreaming apt with two bedrooms, bath, city view, walk to everything, supermarket nearby, get Path.		Positive
…		
I try to like with each paycheck, try to put a little in saving.	Saving and money	Positive
Accumulation process does not look that saving a lot, but can add up over time.		Positive
I do not touch unless it is really important for me to spend.		Positive
Investing early definitely. Then I had lots of money.		Positive
Starting saving as soon as possible.		Positive
Money gives me health and wealth, help a lot of poor people. Most people need money, they will not be sleeping the street.		Positive
I want to have enough money that I could live comfortably.		Positive
…		
I have student’s loan. I guess it worth education. But it depends.	Education	Neutral
I definitely say education is important.		Positive
Worth education? Really depends on college. But I do not think so.		Negative
Colleges are not for everyone because of student’s loan.		Negative
The debt issue is becoming too large. Paid off their debt is stupid.		Negative
I had scholarship and education worth it.		Positive
…		
Celebrities overpay? I do think they are just because of the tax thing. I think that’s severely not ok to make such much money and have such little taxes. That is not fair.	Fairness	Negative
I do not have favorite celebrity. They are definitely overpaid. Considerate the amount of work, talent, it is fair.		Positive
People of color like black, Latinx, Asians, who are obviously coming here for a better life are not getting enough community or government help.		Negative
…		

In Table 7, analysis of these 12 categories reveals positive, negative, or neutral attitudes. Transcript data in the categories of “Family and Marriage,” “Friendship and Relationship,” “Trust and Charity,” and “Saving and Money” show predominantly positive responses. This suggests that relationships, trust, and expectations are highly valued and relatively satisfactory. Conversely, “Wellbeing and Health,” “Rent, Price and Housing,” and “Fairness” show more negative attitudes, indicating that personal wellbeing, social justice, and quality of life are regarded as important but are deemed insufficiently met. For “Work-life balance,” “Job and Retirement,” “Sharing,” “Equality,” and “Education,” respondents’ feelings vary considerably; satisfaction and dissatisfaction coexist. These results suggest that there is substantial room for progress in social sustainability at the individual level and in everyday life.

#### 4.2.2 Axial coding analysis

The second step involves axial coding to extract themes and elements. Because the original transcripts are extensive, it was necessary to identify themes from complex data sets and then recombine them by linking the initial categories. A manual method, combined with a structured thematic analysis (King, 2004), was used to extract themes and elements. Based on Maslow’s hierarchy of needs (Abraham, 1943), the extracted themes and elements were compared with the five levels of needs (Table 8).

**Table 8:** Principal axis coding analysis and extracted theme and factors.

Category	Extracted factors	Extracted theme	Hierarchy of needs
Family and marriage	Acceptance, Belongingness	Micro-social inclusion	Social needs
Friendship and relationship	Social network, Cohesion	Individual social capital	
Trust and charity	Trust, Reciprocity, Sharing	Individual social capital	Security needs, social needs
Sharing	Security dependency		Esteem needs
Saving and money	Quality of life	Micro-social inclusion	Physiological needs
Wellbeing and health	Life related social resources	Individual social capital	Security needs
Rent, price, and housing	Sustainable living and working	Individual social sustainability	
Work-life balance	Sustainable community		
	Social wellbeing		
Job and retirement	Security protection	Micro-social inclusion	Physiological needs
Education	Life related social resources	Individual social capital	Social needs
	Sustainable living and working	Individual social sustainability	Self-actualization needs
	Secure future		
Fairness, equality	Diversity, Cross-cultural	Multicultural background	Social needs
	Acceptance	Micro-social inclusion	Esteem needs
	Reciprocity	Individual social capital	Self-actualization needs
	Equal opportunity	Individual social sustainability	Esteem needs

From Table 8, specific elements emerge from the categories, resulting in meaningful factors. Further, these categories, elements, and themes align with the framework of the revised conceptual model of social sustainability: “multicultural background,” “micro-level social inclusion,” “individual social capital,” and “individual social sustainability.” These extracted elements and themes also fit well with Maslow’s needs, thereby verifying, and refining the variables in the model. The extracted themes reinforce the paper’s focus on micro-level factors that promote social sustainability, indicating that the categories, elements, and themes from the raw transcripts are not only closely related to individual needs but also consistent with the attributes of micro-level social inclusion, individual social capital, and individual social sustainability.

## 5 Conclusion

This paper examines issues of social sustainability in a multicultural context. Multiculturalism has developed over the years as a theory, a political idea, and a social policy. The literature system and the text of street interviews in this paper show that multiculturalism has a wide range of influences and challenges on individual behavior, interactions in everyday life, identity, and social inclusion. Having clarified the conceptual definitions of micro-level social inclusion, individual social capital, and social sustainability, it proposes a preliminary conceptual model of social sustainability. Two research phases were conducted to refine and validate this initial model.

In Phase 1, the paper reviewed published studies on how social inclusion promotes social sustainability, exploring the relationships among social inclusion, social capital, and social sustainability. Findings reveal that while extensive research already addresses social inclusion, the emphasis on how it influences individual wellbeing and quality of life suggests that micro-level social inclusion aligns well with individual social capital, exerting an impact on individual-level social sustainability.

As its theoretical innovation, the final revised conceptual model of social sustainability integrates both cognitive (cultural identity) and behavioral (cross-cultural communication) elements of multiculturalism. Drawing on Maslow’s hierarchy of needs, five fundamental elements were extracted for micro-level social inclusion (equal empowerment, acceptance, belonging, security protection, quality of life), individual social capital (sharing, trust and reciprocity, social cohesion, security dependency, life-related social resources), and individual social sustainability (social wellbeing, sustainable community, equal opportunity, secure future, sustainable living and working). This logically confirms that social inclusion promotes social sustainability through social capital.

In Phase 2, based on short video transcripts, the paper uses preliminary categorization and axial coding to extract elements and themes from original responses, aligning them with the five levels of Maslow’s hierarchy of needs. The 12 categories (Family and Marriage, Friendship and Relationship, Trust and Charity, Sharing, Saving and Money, Wellbeing and Health, Rent, Price and Housing, Work-life balance, Job and Retirement, Education, Fairness, Equality) match the conceptual elements formed in Phase 1, confirming that micro-level social inclusion, individual social capital, and individual-level social sustainability constitute fundamental needs for individual welfare. This validates the variable relationships in the revised social sustainability conceptual model.

## 6 Theoretical contributions and implications

This study contributes in two main ways: conceptualizing micro-level social inclusion and proposing a new social sustainability conceptual model. Researchers and policymakers alike can apply these contributions to various studies and practices.

The first contribution is the concept of micro-level social inclusion. Here, a new definition and element set for micro-level social inclusion are offered. This new conceptual structure centers on micro-level attributes, including processes that support individuals’ social participation, welfare policies granting access to food, housing, health, education, and employment, along with individual capacity, opportunities, dignity, acceptance, and support. This differs from typical discussions of social inclusion that are macro-policy-focused, though micro and macro social inclusion are interlinked. By clarifying the multidimensional nature of micro-level social inclusion, this study provides a conceptually well-defined yet flexible basis for further research examining social inclusion and related factors at different levels (Licsandru and Cui, 2018). The five dimensions extracted for micro-level social inclusion can be operationalized in future research to measure micro-level social inclusion, facilitating broader empirical studies.

The second contribution is the conceptual model of social sustainability. Starting with a preliminary conceptual model, this paper refines the relationships among social inclusion, social capital, and social sustainability, ultimately presenting a revised model. Prior literature typically discusses only social inclusion and social capital (Stanley et al., 2010) or social inclusion and social sustainability (Kohon, 2018). After introducing a multicultural background, the new model fully depicts how cultural identity, cross-cultural communication, micro-level social inclusion, individual social capital, and individual social sustainability interconnect. This provides a foundation for subsequent hypotheses, variable selection, and measurement research.

In the model proposed in this paper, the mediating role of social capital can be summarized as a chain: social inclusion policy, individual social capital accumulation, social network expansion, reciprocal norm reinforcement, and trust accumulation can be enhanced. This mediating role of social capital has been verified in the literature from the perspective of Italian cases (Putnam, 1992) and the interviews in this paper. Individual social capital is the “invisible social infrastructure” that connects micro-social inclusion and social sustainability, and its intermediary value lies in the fact that in the short term, social capital translates social inclusion into concrete cooperative actions, and in the long run, it will form a positive feedback loop of social inclusion, trust and cooperation, and social sustainability.

Practically, this research highlights how micro-level social inclusion, individual social capital, and individual-level social sustainability contribute to advancing social equity, equal opportunities, and long-term social stability and development, thereby enhancing individuals’ wellbeing and quality of life—supported by the interview-based text analysis. Specifically, several focal themes in the interviews offer practical insights into promoting social sustainability:

### 6.1 Personal finance and consumption behavior

Interview discussions addressed how financial education and support can help vulnerable populations (e.g., low-income families and minorities) better manage resources, thereby reducing economic inequality and strengthening social inclusion. Improving financial literacy among these groups fosters sustainable economic development and reduces social disparities, laying a stronger foundation for long-term social stability.

### 6.2 Rent and housing pressure

Safeguarding housing rights is essential for social inclusion. Interviews touched on how policy and market regulations can ensure that all society members, especially low-income groups, have access to affordable housing. Stable housing lessens social inequality, enhances social stability, and supports sustainable urban development.

### 6.3 Work-life balance and financial management

Widespread adoption of work-life balance policies helps ensure all social strata can maintain employment income while enjoying family and leisure time, thereby improving social inclusion. Fostering healthier work-life balance reduces burnout, heightens productivity and life satisfaction, and supports sustainable economic growth.

By examining these interview themes, this paper clarifies that social inclusion, social capital, and social sustainability are not merely interlinked concepts but can be significantly advanced through concrete policy and practice. Such connections are crucial to building a fairer, more sustainable society.

### 6.4 Friendship and financial relationships

Interviews revealed how money can affect friendships across different socioeconomic backgrounds and explored ways to reinforce social support systems, thus encouraging interaction and inclusion among different groups. Healthy social relationships and networks bolster social cohesion and reduce social isolation, promoting stability and sustainable social development.

## 7 Limitations and further research

While this study yields some groundbreaking findings in both theory and practice, it also has clear limitations. Theoretically, although this paper establishes a theoretical model on how social inclusion, social capital, and social sustainability are interrelated, it does not include empirical analyses to test these proposals, leaving the door open for future studies to provide evidence and validate the model. Regarding the interviews, the random street interviews covered broad topics lacking tight focus on the research issues; thus, some randomness inevitably affects the analysis. In terms of systematic literature research, the literature sources of this paper are limited by the limitation of literature data collection software, and there is no extensive collection of literature in various databases, which limits the extensiveness of the research conclusions of this paper.

This research is exploratory, referencing existing studies as examples to build a complete social sustainability model, yet it does not conduct further qualitative or quantitative testing in other related applications. The limitations of this study are obvious, and future research needs to further quantify the relationship between micro social inclusion, individual social capital and social sustainability, and study the nodes of policy optimization and intervention from the perspective of the correlation between social capital index and social sustainability index.

Although the most striking or core proposition of this paper is that social inclusion at the micro level positively affects social capital and drives social sustainability, this paper argues that exploring the negative effects of excessive social inclusion is also a concern for future research. For example, social inclusion is neither unconditional nor cost-free, and the conflict between individual freedom and social norms suggests that there are boundaries to social norms. In practice, excessive social inclusion can lead to controversy, and excessive emphasis on diversity and differentiation also carries the risk of social differentiation.

Future endeavors might test, understand, and refine the proposed model and its applications in managing social sustainability. Potential directions include examining how micro-level social inclusion affects individual-level social sustainability through individual social capital under diverse multicultural themes; clarifying beneficial vs. adverse effects; strategies for amplifying positive impacts while minimizing negatives; and further investigating the micro-level social inclusion concept and its dimensions.

## Data Availability

Interview data for this article can be found online at https://youtube.com/@wefireapp?si=v6mxao3B8625qE0H.
